# Age Differentiation within Gray Matter, White Matter, and between Memory and White Matter in an Adult Life Span Cohort

**DOI:** 10.1523/JNEUROSCI.1627-17.2018

**Published:** 2018-06-20

**Authors:** Susanne M.M. de Mooij, Richard N.A. Henson, Lourens J. Waldorp, Rogier A. Kievit

**Affiliations:** ^1^Department of Psychology, University of Amsterdam, 1018 WB Amsterdam, The Netherlands and; ^2^MRC Cognition and Brain Sciences Unit, Cambridge CB2 7EF, United Kingdom

**Keywords:** aging, differentiation, gray matter, SEM trees, structural equation modeling, white matter

## Abstract

It is well established that brain structures and cognitive functions change across the life span. A long-standing hypothesis called “age differentiation” additionally posits that the relations between cognitive functions also change with age. To date, however, evidence for age-related differentiation is mixed, and no study has examined differentiation of the relationship between brain and cognition. Here we use multigroup structural equation models (SEMs) and SEM trees to study differences within and between brain and cognition across the adult life span (18–88 years) in a large (*N* > 646, closely matched across sexes), population-derived sample of healthy human adults from the Cambridge Centre for Ageing and Neuroscience (www.cam-can.org). After factor analyses of gray matter volume (from T1- and T2-weighted MRI) and white matter organization (fractional anisotropy from diffusion-weighted MRI), we found evidence for the differentiation of gray and white matter, such that the covariance between brain factors decreased with age. However, we found no evidence for age differentiation among fluid intelligence, language, and memory, suggesting a relatively stable covariance pattern among cognitive factors. Finally, we observed a specific pattern of age differentiation between brain and cognitive factors, such that a white matter factor, which loaded most strongly on the hippocampal cingulum, became less correlated with memory performance in later life. These patterns are compatible with the reorganization of cognitive functions in the face of neural decline, and/or with the emergence of specific subpopulations in old age.

**SIGNIFICANCE STATEMENT** The theory of age differentiation posits age-related changes in the relationships among cognitive domains, either weakening (differentiation) or strengthening (dedifferentiation), but evidence for this hypothesis is mixed. Using age-varying covariance models in a large cross-sectional adult life span sample, we found age-related reductions in the covariance among both brain measures (neural differentiation), but no covariance change among cognitive factors of fluid intelligence, language, and memory. We also observed evidence of uncoupling (differentiation) between a white matter factor and cognitive factors in older age, most strongly for memory. Together, our findings support age-related differentiation as a complex, multifaceted pattern that differs for brain and cognition, and discuss several mechanisms that might explain the changing relationship between brain and cognition.

## Introduction

To understand healthy aging, we must understand the relationship between brain changes and cognitive changes. Although much is known about changes in individual measures such as brain volume or memory performance, less is known about age-related changes in the interrelations between neural and cognitive measurements. The “age differentiation” hypothesis describes changes in the organization of cognitive abilities, where differentiation is defined as a low-covariance relationship among abilities or factors (Spearman, 1927; Deary and Pagliari, 1991; Hülür et al., 2011; Blum and Holling, 2017). As people age, there is considerable evidence that they display a loss of differentiation, where cognitive abilities become more correlated, known as “dedifferentiation” (Garrett, 1946; Baltes and Lindenberger, 1997; Ghisletta and Lindenberger, 2003; de Frias et al., 2007). However, evidence for this age differentiation–dedifferentiation hypothesis is mixed: some studies observe a pattern of increase in differentiation followed by dedifferentiation (Li et al., 2004), a meta-analysis observed a weak but significant differentiation effect with age (Blum and Holling, 2017), whereas others observe no change in differentiation (Deary et al., 1996; Juan-Espinosa et al., 2002; Zelinski and Lewis, 2003; Tucker-Drob, 2009; Molenaar et al., 2017). These differences may partly reflect differences in analytical methods, cohorts, and sample sizes (Molenaar et al., 2010).

Even less is known about changes in brain organization as captured by structural covariance, meaning the extent to which regional brain structures covary across individuals (for brain function, see Park et al., 2004; Mechelli et al., 2005; Alexander-Bloch et al., 2013b). Previous studies have demonstrated that measures of structural covariance show similarities with structural connectivity and resting-state functional connectivity (Damoiseaux and Greicius, 2009; Honey et al., 2009; Seeley et al., 2009; Alexander-Bloch et al., 2013b; Tsang et al., 2017; but see Di et al., 2017) as well as with developmental trajectories (Zielinski et al., 2010; Alexander-Bloch et al., 2013a). Despite this interest, few studies have used principled methods to investigate whether age-related differentiation or dedifferentiation occurs for neural measures such as gray matter volume (GMV) and white matter (WM) microstructure. One notable exception is the work by Cox et al. (2016), who found that a single factor for white matter became more prominent with increasing age, suggesting age dedifferentiation. A final open question is whether age differentiation or dedifferentiation occurs not just within neural or cognitive domains, but also between brain and cognition, such that psychological factors become more or less strongly associated with brain structure across the life span.

Understanding the process of age differentiation is crucial for theories of cognitive development and aging. Older adults may display changes in cognitive strategies: for instance, older individuals may rely more on perceptual salience rather than attentional focus, likely due to poorer internal cues (Lindenberger and Mayr, 2014). Within the neural domain, changes in covariance may reflect a range of important biological processes, including adaptive reorganization (Cabeza et al., 2002; Greenwood, 2007; Park and Reuter-Lorenz, 2009), regional (Gianaros et al., 2006) or global (Cox et al., 2016) vulnerability to disease states, accumulating structural consequences of life span functional connectivity (Seeley et al., 2009), and/or the emergence of subgroups that differ in the extent to which they display these patterns.

If age-related changes in cognitive strategy help to counter neural decline, then such strategies may eventually induce a more diffuse covariance pattern. For instance, theories of functional plasticity (Greenwood, 2007) and cognitive reserve (Whalley et al., 2004) suggest that adaptive reorganization in old age leads to decreased covariance between brain structure and cognitive performance. Conversely, theories such as brain maintenance, where preserved cognitive functioning is directly related to maintained brain capacity (Nyberg et al., 2012), do not predict age-related changes in brain–cognition covariance.

Here we examine age differentiation in a large, healthy, population-derived sample (age range, 18–88 years; Cam-CAN, Shafto et al., 2014), using multigroup structural equation modeling (SEM) and SEM trees. To the best of our knowledge, this is the first study to simultaneously examine age differentiation and dedifferentiation of gray matter, white matter, and cognitive factors.

## Materials and Methods

### 

#### 

##### Participants.

As part of a phase 2 trial of the Cambridge Centre for Ageing and Neuroscience (Cam-CAN), data on a wide range of lifestyle, cognitive, and neural tests was collected from a healthy, population-based human adult sample, described in more detail in the study by Shafto et al. (2014). Exclusion criteria include low Mini–Mental State Examination score (≤24), poor hearing (failing to hear 35 dB at 1000 Hz in either ear), poor vision (below 20/50 on the Snellen test), poor knowledge of English (non-native or nonbilingual English speakers), self-reported substance abuse, an indication by the participant's primary care physician that participation would not be appropriate, and serious health conditions that affect participation (e.g., self-reported major psychiatric conditions, current chemotherapy/radiotherapy, or a history of stroke). We also excluded people with MRI contraindications including disallowed implants, pacemakers, recent surgery or any previous brain surgery, current pregnancy, facial or very recent tattoos, or a history of multiple seizures or fits) as well as comfort-related contraindications (e.g., claustrophobia or self-reported inability to lie still for 1 h). A total of 707 people was recruited for the cognitive assessment (359 females and 348 males) including ∼100 individuals from each decile (age range, 18–88 years; mean = 54.63 years; SD = 18.62 years); usable gray matter was collected from 651 people, and white matter from 646 people, sample sizes that are sufficient for moderately complex structural equation models (Wolf et al., 2013). Ethical approval for the study was obtained from the Cambridgeshire 2 (now East of England-Cambridge Central) Research Ethics Committee. Participants gave full informed consent. The raw data to reproduce all analyses can be acquired through the Cam-CAN data portal (https://camcan-archive.mrc-cbu.cam.ac.uk/dataaccess/index.php).

##### Gray matter.

**Figure 1.**
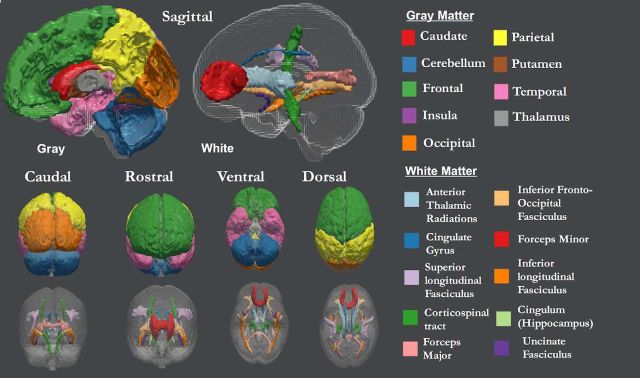
Nine gray and ten white matter tracts, as defined by Montreal Neurological Institute (Mazziotta et al., 2001) and Johns Hopkins University white-matter tractography atlas (Hua et al., 2008).

To examine gray matter (GM) structure, we estimated gray matter volume (GMV) based on the combined segmentation and normalization of 1 mm^3^, T1-weighted, and T2-weighted MR images. For more detail on the preprocessing pipeline, see Taylor et al. (2017). We here use GMV for nine ROIs, as defined by the Montreal Neurological Institute (Mazziotta et al., 2001). This atlas captures a set of canonical gray matter structures and has a similar number of ROIs (9 vs 10) as our white matter measure (see below), allowing us to compare evidence for differentiation or dedifferentiation across gray matter and white matter using models of comparable complexity. The nine ROIs in the MNI atlas are caudate, cerebellum, frontal lobe, insula, occipital lobe, parietal lobe, putamen, temporal lobe, and thalamus (Fig. 1).

##### White matter.

To investigate covariance in white matter (WM) structure, we estimated fractional anisotropy (FA) values in a set of white matter ROIs. FA is a measure of the diffusivity of water molecules that is thought to reflect fiber density, axonal diameter, and myelination. It is also sensitive to age-related changes in cerebral myelin (Kochunov et al., 2012), although there is discussion on the challenges and limitations of FA (Jones and Cercignani, 2010; Jones et al., 2013; Arshad et al., 2016; Wandell, 2016). We computed the mean FA for 10 ROIs, as defined by the Johns Hopkins University white-matter tractography atlas (Fig. 1; Hua et al., 2008): anterior thalamic radiations (ATRs), cerebrospinal tract (CST), dorsal cingulate gyrus (CING), ventral CING (CINGHipp), forceps major (FMaj), forceps minor (FMin), inferior fronto-occipital fasciculus (IFOF), inferior longitudinal fasciculus (ILF), superior longitudinal fasciculus (SLF), and the uncinate fasciculus (UNC). For further details on the white matter pipeline, see the study by Kievit et al. (2016).

##### Cognitive tasks.

Five cognitive tasks were used to assess cognitive processing across the following three broad cognitive domains: language, memory, and fluid intelligence. Language was measured using the following two tasks: (1) the Spot-the-Word test (Baddeley et al., 1993), in which word–nonword pairs (e.g., “daffodil–gombie”) are presented and the participant has to decide which is the real word; and (2) a proverb comprehension test, in which participants were asked to provide the meaning of three common proverbs in English (e.g., “Still waters run deep”), yielding a score between 0 and 6. Our measure of fluid intelligence was the standard form of the Cattell Culture Fair, Scale 2 Form A (Cattell, 1971). This pen-and-paper test contains four subsets with different types of abstract reasoning tasks, namely matrices, series completion, classification, and conditions. Finally, the third domain memory was assessed using measures of immediate and delayed (after 30 min) story recall, as well as recognition, from the logical memory subtest of the Wechsler Memory Scale, Third UK edition (Wechsler, 1997).

##### SEM analyses.

To improve convergence, before the SEM analyses, the neural and cognitive measures were scaled to a standard normal distribution. We used full information maximum likelihood estimation and robust maximum likelihood estimator with a Yuan-Bentler scaled test statistic to account for violations of multivariate normality. To ensure that possible outliers did not affect the results, we fit the models with both full data and data treating univariate outliers (*z*-scores >4 or −4) as missing. Doing so did not meaningfully affect any model comparison, so we report the results for the full dataset. We used SEM to test for evidence for neural and cognitive age differentiation or dedifferentiation in the following three steps: (1) establish an appropriate measurement model; (2) examine adult life span patterns of the factor scores; and (3) formally test for age differentiation or dedifferentiation using multigroup confirmatory factor analysis (MGCFA) and SEM trees (see below for more detail).

All models were fit using the package lavaan (Rosseel, 2012) in the statistical software R (R Core Team, 2016). We assessed overall model fit using the χ^2^ test, root mean square error of approximation (RMSEA) and its associated confidence interval, comparative fit index (CFI), and standardized root mean square residual (SRMR; Schermelleh-Engel et al., 2003). We considered good fits to be as follows: RMSEA < 0.05 (0.05–0.08 is acceptable); CFI > 0.97 (0.95–0.97 is acceptable); and SRMR < 0.05 (0.05–0.10 is acceptable). For the MGCFA, we compared models directly with the likelihood ratio test, the Akaike information criterion (AIC), the Akaike weights test (Wagenmakers and Farrell, 2004), and the sample size-adjusted Bayesian information criterion [saBIC (with associated Schwarz weights)]. For all age comparisons, we defined three discrete, equally sized subgroups: young, middle, and old (Table 1). For each life span multigroup comparison, we compared a model where factor covariance was equality constrained across the three age groups to a model where they were freely estimated. In the constrained model, all parameters were constrained among the groups, except for the means of the factors (to allow for age-related declines). By comparing these nested models, we could determine whether there is evidence for changing factor covariance structure across the life span.

**Table 1.** Demographics of age groups (young, middle, and old) for neural and cognitive measures

	Age group	Sample size (*N*)	Mean age (years)	SD age
Gray matter	Young	217	32.82	6.92
	Middle	217	54.56	6.25
	Old	217	75.56	5.91
White matter	Young	215	32.87	6.89
	Middle	216	54.69	6.32
	Old	215	75.66	5.85
Cognition	Young	235	33.10	7.19
	Middle	236	54.86	6.37
	Old	236	75.84	5.87

In cases where the likelihood ratio test yielded evidence for age differentiation, we visualized the differences by using a technique inspired by local structural equation models (Hildebrandt et al., 2009; but, see also Hülür et al., 2011). This technique allows us to visualize age gradients in model parameters of the covariance structure in a more continuous manner, rather than creating age groups. To do so, we estimated the covariance between factors using a series of age-weighted SEMs for the CFA models with subsets of the sample (*N* = 260 for WM; *N* = 300 for GM, due to estimation variability) in 1 year steps from 18 to 88 years. Next, a kernel function was used to weigh and smooth the observations according to the age gradients (Hildebrandt et al., 2009). The following bandwidth (bw) of the kernel function was used to smooth the age-weighted samples:

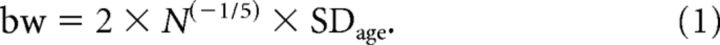
 Visualizing factor covariance allowed for the identification of life span patterns including differentiation and dedifferentiation. If the data are in line with age differentiation, we expect to find that the nested multigroup model with the freely estimated covariance structure is preferred, in such a way that the older subgroup has lower covariance between factors. Evidence for age dedifferentiation would suggest a preference for the freely estimated model, but with higher covariance between the factors in the older subgroup. We first examine differentiation within each domain (gray matter, white matter, and cognition), and finally examine brain–cognition covariance differences. Finally, we used SEM trees, which combine the strength of SEM and decision trees (Brandmaier et al., 2013, 2016). SEM trees partition a dataset repeatedly into subsets based on some covariate of interest to examine whether a likelihood ratio test suggests sufficient evidence of significantly different parameter estimates in each possible subgroup. This method allows us to find covariates and covariate interactions that predict differences in model parameters (in observed and latent space) in a hierarchical fashion. The addition of SEM trees to the multigroup analyses enables us to analyze age in a continuous nature and trace potential age differences in optimal splits. In this study, SEM trees were used to investigate whether the covariance structure in the same neural and cognitive factors model as used in the multigroup SEM models changed with age. According to the differentiation hypothesis, SEM trees would split the dataset into subsets with different covariance structures according to the continuous covariate age.

All SEM trees were analyzed with the package “SEM Trees” (Brandmaier et al., 2013) in R using the OpenMx package for SEM. We imposed the same models as with the multigroup SEM to compare the results in favor of or against the differentiation hypothesis. All paths were constrained, except for the covariance between the factors and the factor means to allow age-related decline, but since the factor means change alongside the covariance, the source of the potential split is rather ambiguous. Notably, this technique allows for the specification of focal parameters, such that only differences in model fit due to these key parameters are used to partition the data into subsets. Here, we base possible splits only on the factor covariance, as these splits reflect the age differentiation hypothesis. The criterion for best split is based on a Bonferroni-corrected likelihood ratio test of differences between the groups resulting from a given split (Brandmaier et al., 2013). To ensure a sufficient number of participants given model complexity, we only allowed splits where the minimal sample per subgroup would be at least 200 participants.

## Results

### Gray and white matter covariance

**Figure 2.**
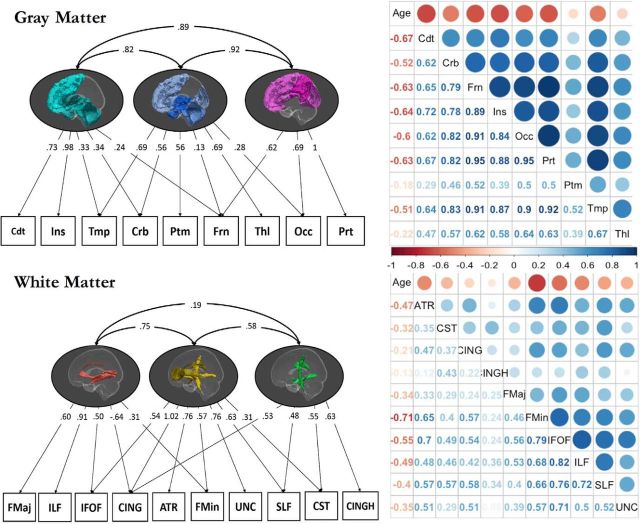
The three-factor model for gray matter (top left) underlies the following nine ROIs: caudate (Cdt), insula (Ins), temporal (Tmp), cerebellum (Crb), putamen (Ptm), frontal (Frn), thalamus (Thl), occipital (Occ), and parietal (Prt). The three-factor model for white-matter (bottom left) underlies the following 10 ROIs: FMaj, CING, IFOF, ILF, ATRs, FMin, UNC, SLF, CST, and CINGH. The darker colors in the lateral brain views represent the regions with the highest factor loadings. Path coefficients are fully standardized. The correlation matrices are shown for gray matter (top right) and white matter (bottom right), along with age.

**Figure 3.**
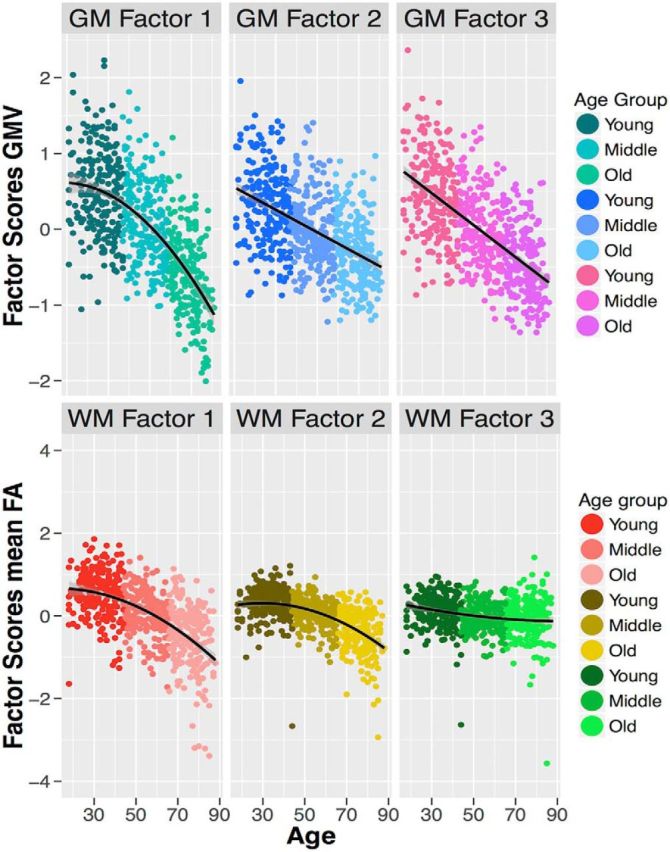
Age-related decline in gray matter volume (top three plots) and white matter FA (bottom plots) factor scores, according to the age groups (young, middle, and old) with best functional form shown (linear or nonlinear). William's test for dependent correlations showed that the effects of age were significantly different: across the gray matter ROIs: *t*_cor1_2(651)_ = −9.46, *p* < 0.001; *t*_cor1_3(651)_ = −2.14, *p* = 0.033; *t*_cor2_3(651)_ = 12.79, *p* < 0.001; and across white matter ROIs: *t*_cor1_2(646)_ = −12.07, *p* < 0.001; *t*_cor1_3(646)_ = −12.07, *p* < 0.001; *t*_cor2_3(646)_ = −8.28, *p* < 0.001.

To specify a measurement model amenable to multigroup confirmatory factor analysis, we first examined a plausible candidate model using an exploratory factor analysis (EFA). For gray matter, we established that a three-factor solution was preferred. This three-factor model showed adequate fit in the following CFA analysis: χ^2^ (19) = 82.384, *p* < 0.001, RMSEA = 0.072 [0.057–0.087], CFI = 0.990, SRMR = 0.016. For white matter, a three-factor model showed marginally acceptable fit: χ^2^ (26) = 133.897, *p* < 0.001, RMSEA = 0.080 [0.068–0.093], CFI = 0.966, SRMR = 0.025. The measurement models are shown in Figure 2, along with their correlation matrices. As the precise factors will depend to some extent on the atlas used, we will not label the factors, but will examine the covariance patterns in more detail. The first gray matter factor (Fig. 2, teal) is characterized by strong loadings especially on the insula. The second gray matter factor (Fig. 2, blue) is characterized by a relatively broad set of medium-sized factor loadings, with an especially strong factor loading for temporal and thalamic gray matter volume. The third gray matter factor (Fig. 2, pink) is characterized most strongly by parietofrontal covariance. Although a single-factor model fits poorly for gray matter, the correlations among gray matter factors are relatively strong, especially compared with the white matter factors, which are more globally differentiated. The white matter measurement model also yields three factors. The first white matter factor (Fig. 2, red) is characterized by strong loadings on more posterior ILF and FMaj tracts, and a negative factor loading on the cingulum. The second white matter factor (Fig. 2, yellow) is characterized most strongly by the cingulum, but has a broad set of positive factor loadings across the majority of tracts. Finally, the third factor (Fig. 2, green) loads most strongly on the ventral cingulum. The effects of age on the factor scores are shown in Figure 3, revealing different effect sizes, as well as different functional forms (linear and nonlinear).

First, we tested for age differentiation using an MGCFA, where the population is divided into three age groups with equal sample sizes: young, middle, and old (Table 1). This group-level comparison tests for age-related differences in specific parameters, while constraining the rest of the model (Δdf = 6, as either three- or nine-factor covariances were estimated). Although constraints will generally lead to poorer model fit overall, we were interested in the specific comparison between the two nested models that represent age differentiation versus no differentiation. Fitting these two models, we found that for the gray matter factors, a model where factor covariances were estimated freely across age groups showed better fit: Δχ^2^ (6) = 19.591, *p* = 0.003 (Table 2). Akaike weights showed that the freely estimated model (with age-varying factor covariance) was 696 times more likely to be the better model given the data (Wagenmakers and Farrell, 2004). Using the same procedure for white matter, we found that the model with the freely estimated covariance also showed better fit: Δχ^2^ (6) = 25.430, *p* = 0.001, Akaike weights = 6297 in favor of the freely estimated model.

**Table 2.** Model fit indices within white and gray matter, where the model with freely estimated covariance structure was preferred for both. *w_i_*(AIC) = the rounded Akaike weights; *w_i_*(BIC) = the rounded Schwarz weights

	Model	df	AIC	*w_i_*(AIC)	saBIC	*w_i_*(saBIC)	χ^2^	Δχ^2^	Δdf	*p* value
Gray matter	Freely estimated	115	8834.1	0.999	8905	0.998	579.79			
	Constrained	121	8847.2	0.001	8911	0.002	604.88	19.59	6	0.003
White matter	Freely estimated	144	14,229	0.999	14,295	0.996	769.83			
	Constrained	150	14,246	0.001	14,305	0.004	799.32	25.43	6	0.001

**Figure 4.**
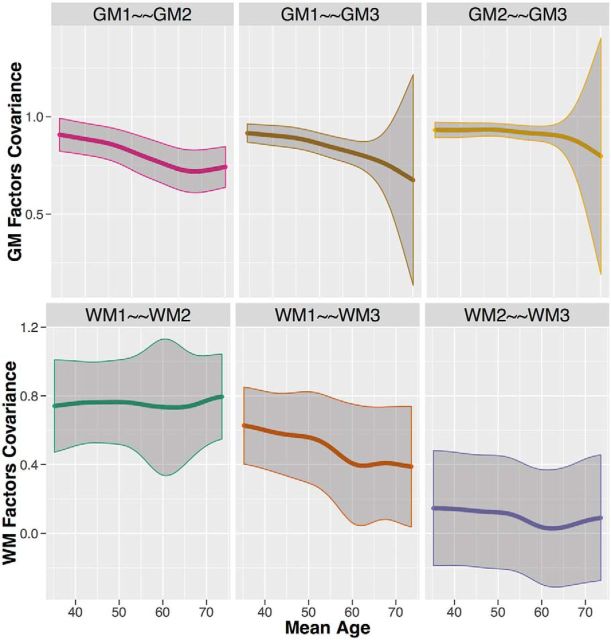
Differences in standardized covariance between the gray matter factors (top) and white matter factors (bottom) with age. The 95% confidence intervals are displayed as the shaded area around the mean.

Next, we visualized the changing covariance within gray and white matter to assess evidence for age differentiation, dedifferentiation, or some other pattern. The top three plots in Figure 4 illustrate the difference in standardized covariance between each pair (GM1–GM2, GM1–GM3, and GM2–GM3) of gray matter factors. The strongest pattern is that factor GM1 displays considerable age differentiation: GM1 becomes more dissimilar to the two other gray matter factors with increasing age. For the white matter factors, the dominant pattern in the bottom three plots of Figure 4 is the differentiation between factors WM1 and WM3, while the standardized covariance between factors WM1 and WM2, and between factors WM2 and WM3, remains relatively stable.

**Figure 5.**
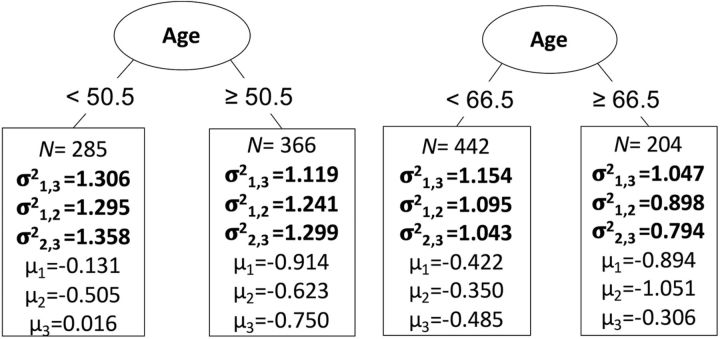
SEM tree analysis with optimal splits for gray matter (left) at the age of 50.5 years, and for white matter (right) at the age of 66.5 years. The standardized factor covariance (σ^2^) and factor means (μ) are depicted per subgroup, including the size of the group.

Finally, we validated the same question using the more exploratory technique of SEM trees with age as continuous covariate. For gray matter, the best split of the sample was given at the age of 50.5 years (χ^2^ = 76.02, df = 3), separating the participants into a young (*N* = 285) and old (*N* = 366) subgroup (Fig. 5, left plot). In line with the MGCFA, this analysis shows that the covariance between the gray matter factors decreases in old age. For white matter, we also find a single optimal split at a much older age of 66.5 years (χ^2^ = 36.07, df = 3), separating participants in a younger (*N* = 442) and older age group (*N* = 204). The factor covariance between the white matter factors decreased in old age similar to gray matter (Fig. 5, right plot). Together, these three analytic strategies converge on the same conclusion: we observe age differentiation, or decreased covariance, among neural factors starting after middle age.

**Figure 6.**
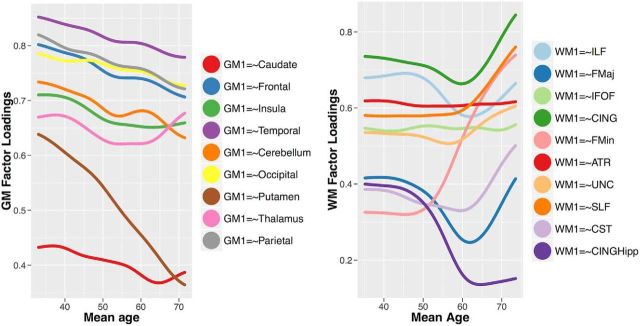
Standardized factor loadings in a one-factor model of gray matter (left) and white matter (right) across the life span.

A recent article by Cox et al. (2016) used a different analytic strategy than ours: instead of focusing on factor covariance, they imposed a single-factor model and examined factor loadings as they changed across the life span. To examine the robustness of our findings to such alternative approaches, we likewise imposed a single-factor model across all brain regions, and tested whether factor loadings, rather than covariance, differ across three age groups (young, middle, and old). For gray matter, even though the one-factor model did not fit well (χ^2^ (27) = 320.516, *p* < 0.001, RMSEA = 0.129 [0.117–0.141], CFI = 0.955, SRMR = 0.026), and a likelihood ratio test showed that it was a worse description of the data than the three-factor model (Δχ^2^ (8) = 227.66, *p* < 0.001), the model with freely estimated factor loadings is again better than the constrained model (Δχ^2^ (16) = 109.27, *p* < 0.001, Akaike weights = 6.13 × 10^22^), supporting differences in gray matter factor loadings across the adult life span. A visual inspection of the smoothed local SEM (LOSEM) shows that all factor loadings decline with age, again in line with age differentiation (Fig. 6). Together, this represents strong evidence for age differentiation for gray matter factors, a pattern that does not depend on the precise analytical method.

For the white matter, we again found that the one-factor model for white matter did not fit well (χ^2^ (35) = 418.652, *p* < 0.001, RMSEA = 0.130 [0.120–0.140], CFI = 0.879, SRMR = 0.062), with the three-factor model showing better fit (Δχ^2^ (9) = 259.23, *p* < 0.001; Table 2). Nonetheless, within the single-factor conceptualization, we again observe that the freely estimated factor loadings were preferred over the constrained version (Akaike weights = 7.90 × 10^28^). The LOSEM plot in Figure 6 shows a complex pattern, with several factor loadings increasing (e.g., forceps minor and superior longitudinal fasciculus), while others remain stable [e.g. inferior fronto-occipital fasciculus (IFOF), anterior thalamic radiations (ATR)] or decline [e.g., hippocampal CING (CINGH)]. The subset of increasing factor loadings is partly in line with the findings of Cox et al. (2016), who suggested age dedifferentiation of white matter tracts as the role of the general factor increases with age. However, the poor fit of the one-factor model and the fact that factor loadings in our sample show evidence for both age differentiation as well as dedifferentiation, suggest that a cautious interpretation is warranted, with further, ideally longitudinal, investigation being crucial to understand the complex age-related differences in white matter covariance.

Finally, we implemented MGCFA on the combination of white and gray matter, with the same measurement models imposed, to see whether the covariance between white and gray matter factors changes across the life span. We did not find evidence for age-related difference in the covariance between WM and GM: the more parsimonious constrained model of the covariance structure was more likely (Δχ^2^ (18) = 24.10, *p* = 0.152). These tests establish that the covariance within neural factors for both gray and white matter is different across the three age groups, but the covariance between the two neural measures does not differ across age groups.

### Cognitive factors

**Figure 7.**
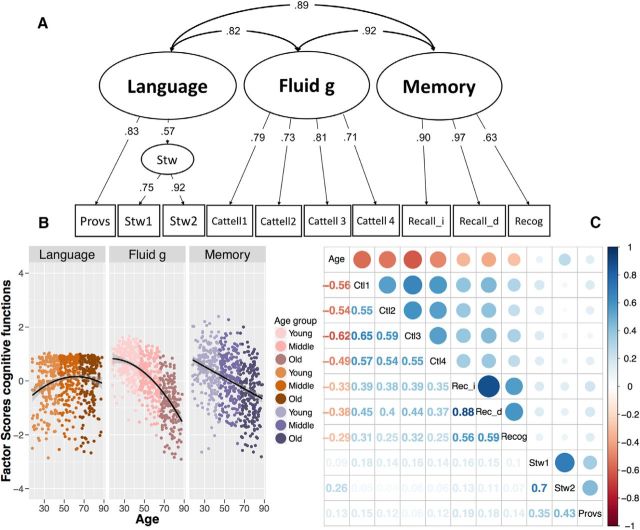
***A***, Confirmatory factor model for cognitive processing based on proverb comprehension (Provs), two spot-the-word tasks (Stw1 and Stw2), four Catell subtests (Catell 1–4) relating fluid intelligence (Fluid g), immediate and delayed recall (Recall_i, Recall_d, respectively), and delayed recognition (Recog). All paths are fully standardized. ***B***, Age-related difference according to the age groups of the three cognitive factors (language, fluid intelligence, and memory) with the best functional form shown (linear or nonlinear). William's test for dependent correlations showed that the effects of age were significantly different: between language and fluid g, *t*_(707)_ = 24.21, *p* < 0.001; between fluid g and memory, *t*_(707)_ = −11.07, *p* < 0.001; and between language and memory, *t*_(707)_ = 12.9, *p* < 0.001. ***C***, Correlation matrices between all cognitive tasks and age.

We next examined age-related differences in covariance across cognitive factors. We defined three latent factors for the measurement model (Fig. 7*A*), based on the following a priori defined cognitive domains: (1) language, modeled by two Spot-the-Words tasks as a first-order factor and a single proverb comprehension task; (2) fluid intelligence, fit to the four scores on Cattell subtests; and (3) memory, fit to immediate recall, delayed recall, and delayed recognition scores. The three-cognitive factor model, shown in Figure 7*A*, fit the data well: χ^2^ (31) = 59.030, *p* = 0.002, RMSEA = 0.036 [0.022–0.049], CFI = 0.988, SRMR = 0.030. The three-factor model fit considerably better than a one-factor solution (Δχ^2^ (4) = 336.43, *p* < 0.001; Akaike weights = 3.31 × 10^273^). Figure 7*B* shows the life span differences in the three cognitive factor scores.

We looked for evidence for age differentiation among the cognitive factors across the three age groups with MGCFA, and found that the constrained covariance model was more likely: Δχ^2^ (6) = 4.984, *p* = 0.546, in line with an absence of either age-related cognitive differentiation or dedifferentiation. When we examined the same question using SEM trees, we did not observe a significant split in covariance structure with age. The lack of evidence for differentiation or dedifferentiation in both methods suggests a relative static covariance structure of cognitive abilities across the life span, contrary to findings in studies by, for example, de Frias et al. (2007), but in line with the findings in the studies by Deary et al. (1996); Juan-Espinosa et al. (2002); and Tucker-Drob (2009).

### Neurocognitive age differentiation

Finally, having examined brain and cognitive differentiation separately, we investigated their interaction to explore differences in brain–cognition covariance across the life span. To do so, we imposed the same measurement models as used above, first for gray matter and cognition, then for white matter and cognition. Our goal was to see whether there is evidence for neurocognitive age differentiation, as indicated by differing covariance between brain structure and cognitive function across the life span.

With MGCFA, we did not find evidence for neurocognitive age differentiation in the covariance of gray matter with cognition: the more parsimonious constrained model of the covariance structure was preferred (Δχ^2^ (18) = 21.53, *p* = 0.253), suggesting a relative stable relationship between the gray matter and cognitive factors across the life span. Similarly, the SEM trees did not show a significant split in the factor covariance with age.

**Figure 8.**
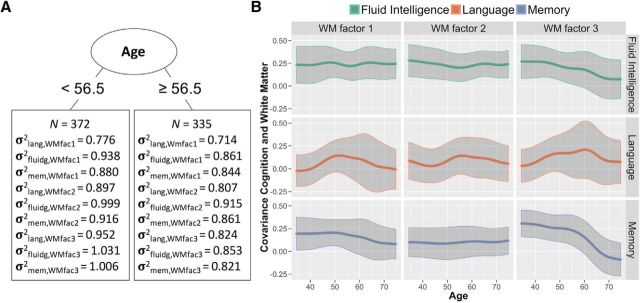
***A***, The optimal age split based on the factor covariance between white matter and the cognitive factors language (lang), fluid intelligence (fluidg), and memory (mem) using SEM trees. ***B***, Differences in the intercorrelations between the cognitive and white matter factors across the life span according to LOSEM. The bottom right shows the one pathway that displays evidence for neurocognitive age differentiation.

For white matter, however, the multigroup analysis suggested that the freely estimated covariance structure was preferred: Δχ^2^ (18) = 37.27, *p* = 0.005, showing age-related differences in the relationship between white matter and cognitive factors. In the SEM tree analysis, we found an optimal split at the age of 56.5 years (χ^2^ = 60.15, df = 9). Notably, all factor covariance in the old age subgroup (*N* = 335) decreased compared with that in the young age subgroup (*N* = 372; Fig. 8*A*).

To examine the source and trend of this neurocognitive age differentiation, we plotted smoothed LOSEM age-weighted measurement models of the nine covariances among the three cognitive and three white matter factors (Fig. 8*B*). Visual inspection suggested that this age-related difference in the relationship between cognition and white matter was driven most strongly by a specific pathway, namely the covariance between WM3 and memory. This visual inspection was confirmed by a *post hoc* test, where a model with a freely estimated covariance between WM3 and memory was strongly preferred over the constrained model: Δχ^2^(2) = 27.34, *p* < 0.001. The covariance between this white matter factor and memory performance declined steadily, especially in old age, suggesting a form of neurocognitive age differentiation. Further *post hoc* comparisons for the other factors were not significant. It is noteworthy that the third white-matter factor was the (only) factor characterized by the CINGHipp, the part of the cingulum that is directly interconnected with the hippocampal formation (Hua et al., 2008; Fig. 2). This suggests a decoupling of memory performance from the white matter networks associated with the hippocampus; an intriguing pattern that we return to below.

## Discussion

In this study, we examined the notion of age differentiation within and between cognitive and neural factors across the adult life span. We found evidence for age differentiation within both GM and WM, such that the covariance between (a subset of) GM factors and the covariance between (a subset of) WM factors is lower in older adults. In contrast, the cognitive factors displayed a stable covariance structure, providing no evidence for differentiation or dedifferentiation. Finally, we observed a specific pattern of age differentiation between WM and cognition, driven almost exclusively by a decoupling between a WM factor highly loading on the hippocampal cingulum and the cognitive factor associated with memory.

For GM, EFA revealed that a three-factor model was preferred. The main effect of age was to reduce the covariance between the first factor (which loaded most on caudate and insula) and the other two factors. This neural differentiation was also observed when imposing a single-factor model, with factor loadings decreasing across the life span. Note that the precise number and nature of factors is likely to depend on the dimensionality of the data. Here we chose a mask characterized by a small number of ROIs (nine in total) to keep the GM model comparable in dimensionality to the WM tracts and cognitive variables. Moreover, a limited number of ROIs was necessary to achieve tractable SEM complexity, given the sample size and subgroup analyses. Nonetheless, our ROIs had sufficient resolution to suggest that distinct networks of those regions differentiate in unique ways, resulting in structural networks that become more dissimilar across individuals in old age.

For WM, a three-factor model of the 10 major WM tracts was also preferred. With this model, we again found evidence for differentiation, with the most noticeable effect being age-related reductions in the covariance between the first factor (which loaded most highly on the inferior fronto-occipital fasciculus and inferior longitudinal fasciculus) and the third factor (which loaded most highly on the ventral cingulum and projection fibers of corticospinal tract). The results from fitting an alternative single-factor model (Cox et al., 2016) were less clear, with both deceases and increases in various factor loadings with age, with the increases suggesting some dedifferentiation. A promising future avenue to better understand this complex pattern of white matter covariance differences is to examine longitudinal changes in white matter covariance, although at present there are few such datasets available.

Several mechanisms might contribute to our findings of differentiation within GM and within WM. First, the differentiation may reflect declines in structural connectivity during healthy aging (Spreng and Turner, 2013). For example, reductions in gray matter covariance may follow reductions in white matter covariance (e.g., myelination) that cause less efficient communication and coactivation between brain regions, over time leading to decreased structural similarity. This is consistent with the present lack of evidence for differentiation between GM and WM. Another possibility is that the differentiation reflects distinct subpopulations of people that diverge across the life span. For instance, if subsets of the older population suffer from medical conditions that differentially affect specific brain regions (e.g., higher blood pressure; Gianaros et al., 2006), this will also lead to a more complex covariance pattern for the older population. Note that it is also possible that systemic age-related effects lead to age-related increases in covariance, or the dominance of a single factor (Cox et al., 2016), which may be disguised by the causes of differentiation described above. Future studies should combine longitudinal imaging approaches with repeated health data to test the plausibility of these explanations in explaining the patterns observed here.

In line with most previous findings (Deary et al., 1996; Juan-Espinosa et al., 2002; Tucker-Drob, 2009), we did not observe evidence for cognitive age differentiation or dedifferentiation, instead finding a stable covariance structure across the life span. More importantly, we examined, for the first time, age differentiation between neural and cognitive factors. Specifically, we observed decreased covariance between a WM factor associated with hippocampal connectivity and a factor associated with memory. This decreased dependency of memory performance on WM integrity may relate to recent analyses of functional connectivity in healthy aging. For instance, Salami et al. (2014) observed greater connectivity within a hippocampal network during rest in older people relative to younger people, but decreased connectivity between the hippocampal network and other cortical networks during mnemonic tasks. Notably, this pattern of “aberrant hippocampal decoupling” (Salami et al., 2014, page 17654) was stronger in individuals with lower white matter integrity near the hippocampus and was associated with poorer memory performance. Westlye et al. (2011) also found aberrant hippocampal functional connectivity associated with poorer performance, and suggested that failures of task-related hippocampal decoupling may elevate the risk of cognitive decline by increasing the metabolic burden on the hippocampus. In a longitudinal structural investigation, Gorbach et al. (2017) observed a robust brain–cognition change–change association between episodic memory decline and hippocampal atrophy in older adults (age range, 60–85 years), which is in line with brain maintenance. Future work integrating longitudinal investigations of the between-individual measurement models across time points in concert with within-subject change–change modeling will be able to reconcile these findings.

An alternative explanation of the decreased covariance between WM and memory observed here is the notion of cognitive reserve (Stern, 2002, 2009; Whalley et al., 2004), which posits that the degree of brain pathology in certain individuals does not directly correspond to the manifestation of cognitive impairment. Certain life span exposures (e.g., high levels of education) are considered protective against cognitive decline. This implies that in older age, as the compensatory mechanisms of cognitive reserve become more prominent, memory performance should depend less on the brain structure, leading to the type of neurocognitive differentiation (i.e., decreased covariance) observed here. However, the precise consequences of cognitive reserve on covariance patterns likely depend on the idiosyncrasies of the sample under investigation. Moreover, it is unclear why we observe a mostly specific pattern of age-related differentiation (between WM and memory), rather than a more general neurocognitive differentiation.

A limitation of our study is that the sample is cross-sectional. The consequence is that, although we can examine age differentiation between individuals, we cannot generalize our findings to intraindividual changes over the life span (Salthouse, 2011). Acquiring longitudinal imaging and cognitive data would allow more detailed investigation of age-related changes in covariance among cognitive and neural factors. Moreover, the recruitment procedure in the Cam-CAN study included two age-correlated selection criteria that may bias the covariance population parameters: the exclusion of participants by general practitioners, and our exclusion of individuals with poor hearing and poor vision for reasons of procedural uniformity. Both hearing and vision are known to correlate with cognition, especially in old age (Baltes and Lindenberger, 1997), so that these procedures induce a positive selection bias of disproportionately healthy individuals in old age. Although age-correlated selection bias will inevitably be present in studies, the degree of bias can be reduced through alternative recruitment procedures such as general registry (de Frias et al., 2007) and/or using more liberal inclusion criteria such as in the Berlin Aging Study (Baltes and Lindenberger, 1997), where subgroups of individuals were blind or deaf or had received a diagnosis of mild dementia. Furthermore, we focus on a relatively limited range of cognitive and neural variables to enable SEMs with a tractable set of parameters. Possible solutions may be found in, for instance, regularized SEM (Jacobucci et al., 2016) that allows measurement and structural models to be based on a larger set of neural and cognitive indicators. Alternatively, larger samples, possibly depending on integration across cohorts, would allow the fitting of higher dimensional measurement models (with possibly an overall better fit) and simultaneously explore generalizability. A second limitation of our study concerns potential differences in data quality across the life span. For instance, if older adults move more, and the effects of this motion of the imaging data cannot be fully accommodated (Geerligs et al., 2017), this may induce a decrease in covariance simply due to less reliable measurement. However, age-related decreases in data quality would seem unlikely to fully explain our findings, given that the pattern of age differentiation was limited to some, but not all, neural factors: increased measurement error in older adults would be expected to produce more uniform decreases in covariance between all pairs of factors.

Our findings show how multigroup confirmatory factor analysis and SEM trees can be powerful techniques for investigating theories of neurocognitive aging, such as age differentiation, allowing researchers to investigate mechanisms of healthy and pathological aging in a flexible yet principled manner. Together, these techniques revealed a complex pattern of age-related differentiation in gray and white matter, but not in cognition, together with a specific differentiation in the relationship between white-matter tracts and memory. Future work on the long-term, developmental patterns of covariance across the life span may help to further elucidate the mechanisms underlying these observations.
